# Subclinical Hypothyroidism and Type 2 Diabetes: A Systematic Review and Meta-Analysis

**DOI:** 10.1371/journal.pone.0135233

**Published:** 2015-08-13

**Authors:** Cheng Han, Xue He, Xinghai Xia, Yongze Li, Xiaoguang Shi, Zhongyan Shan, Weiping Teng

**Affiliations:** Department of Endocrinology and Metabolism, Institute of Endocrinology, Liaoning Provincial Key Laboratory of Endocrine Diseases, the First Affiliated Hospital of China Medical University, Shenyang, Liaoning Province, People’s Republic of China; Harvard Medical School, UNITED STATES

## Abstract

**Background:**

Abundant evidence suggests an association between subclinical hypothyroidism (SCH) and type 2 diabetes mellitus (T2DM), but small sample sizes and inconclusive data in the literature complicate this assertion.

**Objective:**

We measured the prevalence of SCH in T2DM population, and investigated whether T2DM increase the risk of SCH and whether SCH was associated with diabetic complications.

**METHODS:**

We conducted a meta-analysis using PubMed, EMBASE, Web of Science, Wan Fang, CNKI and VIP databases for literature search. We obtained studies published between January 1, 1980 to December 1, 2014. The studies were selected to evaluate the prevalence of SCH in T2DM subjects, compare the prevalence of SCH in T2DM subjects with those non-diabetics, and investigate whether diabetic complications were more prevalent in SCH than those who were euthyroid. Fixed and random effects meta-analysis models were used, and the outcome was presented as a pooled prevalence with 95% confidence interval (95% CI) or a summary odds ratio (OR) with 95% CI.

**RESULTS:**

Through literature search, 36 articles met the inclusion criteria and these articles contained a total of 61 studies. Funnel plots and Egger’s tests showed no publication bias in our studies, except for the pooled prevalence of SCH in T2DM (*P* = 0.08) and OR for SCH in T2DM (*P* = 0.04). Trim and fill method was used to correct the results and five potential missing data were replaced respectively. The adjusted pooled prevalence of SCH in T2DM patients was 10.2%, meanwhile, T2DM was associated with a 1.93-fold increase in risk of SCH (95% CI: 1.66, 2.24). Furthermore, SCH might affect the development of diabetic complications with an overall OR of 1.74 (95% CI: 1.34, 2.28) for diabetic nephropathy, 1.42 (95% CI: 1.21, 1.67) for diabetic retinopathy, 1.85 (95% CI: 1.35, 2.54) for peripheral arterial disease, and 1.87 (95% CI: 1.06, 3.28) for diabetic peripheral neuropathy.

**Conclusions:**

T2DM patients are more likely to have SCH when compared with healthy population and SCH may be associated with increased diabetic complications. It is necessary to screen thyroid function in patients with T2DM, and appropriate individualized treatments in addition to thyroid function test should be given to T2DM patients with SCH as well.

## Introduction

Diabetes is the most common chronic endocrine disease characterized by hyperglycemia resulted from impaired insulin secretion and/or insulin action [1]. Chronic diabetic hyperglycemia is associated with long-term organ damage, dysfunction and failure. Complications, such as vision loss, renal failure and cardiovascular diseases, are often outcomes of diabetes [2–4]. As the population ages and obesity increases, diabetes will increase as well. The global prevalence is predicted to be 11.1% in 2033, affecting 600 million people [5]. China is the world's most populous country, and data from the National Diabetes Prevalence Survey carried out from 2007 to 2008 revealed a prevalence of diabetes in Chinese adults to be 9.7% [6]. Thus, diabetes has reached epidemic proportions worldwide, and this burdens healthcare services and increases healthcare costs [7].

Numerous epidemiological studies indicate the higher prevalence of overt hypothyroidism in type 2 diabetes mellitus (T2DM) population than in the general population[8, 9]. However, the relationship between subclinical hypothyroidism (SCH) and T2DM is controversial. SCH, the slight hypothyroidism state, is asymptomatic but mild elevations in thyroid-stimulating hormone (TSH) with normal circulating free thyroid hormone concentrations are observed [10]. Numerous studies suggest that SCH is associated with hypertension, high cholesterol, and abnormal homocysteine level and patients with SCH have a higher risk of metabolic syndrome, atherosclerosis, cardiovascular events, and mortality [11–13]. Presently, controversy persists about indications for treatment of SCH and whether individuals should be routinely screened for this dysfunction [14, 15].

The guidelines of the American Thyroid Association (ATA) and the European Thyroid Association (ETA) recommend screening thyroid function in patients with Type 1 diabetes annually[16, 17]. However, there is lack of definitive guidance, local policies and/or practices focused on the screening of thyroid dysfunction in T2DM. Furthermore, despite of data suggesting a relationship between T2DM and SCH, conclusions are inconsistent and sample sizes are small. Thus, to investigate the association between SCH and T2DM, and to understand any role of SCH has on diabetic complications, we performed a systematic review and meta-analysis to present a pooled prevalence level of SCH in T2DM patients and to assess the diabetic complications risk in T2DM individuals with SCH.

## Materials and Methods

### Search strategy

In order to find relating articles on prevalence of SCH in T2DM subjects, and articles regarding the prevalence of complications in T2DM patients with SCH, we searched for English articles online using PubMed, EMBASE, and Web of Science. For all the Chinese articles, we searched online using the Chinese National Knowledge Infrastructure (CNKI), Chinese Wanfang and Chongqing VIP databases. All articles were published from January 1, 1980 to December 1, 2014. We adopted the following search strategies: 1. ((((((hypothyroidism [Title/Abstract]) OR TSH [Title/Abstract]) OR thyrotropin [Title/Abstract]) OR thyroid stimulating hormone [Title/Abstract])) AND ((diabetes [Title/Abstract]) OR hyperglycemia [Title/Abstract])) AND prevalence[Title/Abstract]; 2. (((((hypothyroidism [Title/Abstract]) OR TSH[Title/Abstract]) OR thyrotropin [Title/Abstract]) OR thyroid stimulating hormone[Title/Abstract])) AND diabetic complication [Title/Abstract]. To avoid overlooking any relevant study, we also scanned relevant reference lists and reviews to find additional studies. We attempted to contact the authors of the identified papers for necessary data not given in the original texts. Our study was conducted in adherence to the Preferred Reporting Items for Systematic Reviews and Meta-Analyses guidelines (PRISMA) [18], as shown in S1 Checklist.

### Study selection

The study inclusion criteria were: (1) The design of the study had to be observational; (2) All the subjects involved were either T2DM individuals who had been evaluated for thyroid function, or individuals with/without SCH and were enrolled in a clinical and/or laboratory evaluation for diabetic complications;(3) outcome of interest was SCH prevalence in T2DM patients or the prevalence of diabetic complications in T2DM patients with SCH; (4) studies had adequate information for a pooled prevalence or odds ratio analysis; (5) when results obtained from the same population was reported in more than one articles, the most detailed report was included.

On the contrary, studies were excluded if they: (1) were case reports, letters, reviews, editorials, or expert opinions; (2) lacked necessary information such as age and year of study;(3) involved subjects that were pregnant or had chronic renal insufficiency; (4) included diabetics who had type 1 diabetes or unspecified conditions; (5) did not clearly mention the diagnostic criteria of SCH or diabetic complications; (6) included single-gender diabetics;(7) did not use immunochemiluminescent assay(ICMA) as laboratory detection method for thyroid function.

### Data extraction and quality assessment

Two investigators independently conducted the systematic literature search, study selection, data extraction, and quality assessment. Discrepancies were resolved through discussion and consensus between the two investigators. For all included studies, data on the first author’s name, publication year, study year, age, location, the prevalence of SCH in T2DM or non-diabetics, and the prevalence of diabetic complications in SCH patients or euthyroid subjects were extracted.

As reported previously, in order to describe the quality of the researches that were incorporated in our meta-analysis, and to evaluate risk of bias in relation to methodological quality, we assessed the quality of observational researches[19]. The methodological quality of observational case-control studies were appraised by the Newcastle-Ottawa Scale (NOS) [20], and the cross-sectional studies were assessed using the evaluation criteria recommended by Agency for Healthcare Research and Quality (AHRQ) by 2 reviewers as mentioned previously[21].

### Statistical analysis

All analyses were performed using STATA software, version 11.0 (Stata Corp LP, TX). We computed prevalence according to measurements reported previously [22, 23]. Prevalence for SCH in patients with T2DM was estimated as a total number of cases with SCH divided by the number of total patient with T2DM and was reported with 95% confidence interval (95% CI). We then performed a meta-analysis to assess the degree of correlation between T2DM and SCH risk expressed as odds ratio (OR) and 95% CI. Moreover, other meta-analyses were performed to evaluate the risk of diabetic complications in T2DM patients with SCH with ORs and 95% CIs. Heterogeneity among studies was measured with the I^2^ index and *P* value as previously[22]. I^2^ index of 25, 50 and 75% were each considered to be low, moderate and high levels of heterogeneity, respectively. For heterogeneity at moderate or high level, we adopted a random-effects meta-analysis instead of using a fixed-effects model [24]. If any moderate or high heterogeneity existed, we subsequently conducted a subgroup analysis to explore the source of the heterogeneity. Next, we examined publication bias by visual interpretation for funnel plot asymmetry and Egger's test [25]. For Egger’s test, *P* < 0.1 was considered to be statistically significant. Trim and fill analysis was applied if there was publication bias. For other analyses, *P* < 0.05 was considered to be statistically significant.

## Results

### Description of studies

**Fig 1:**
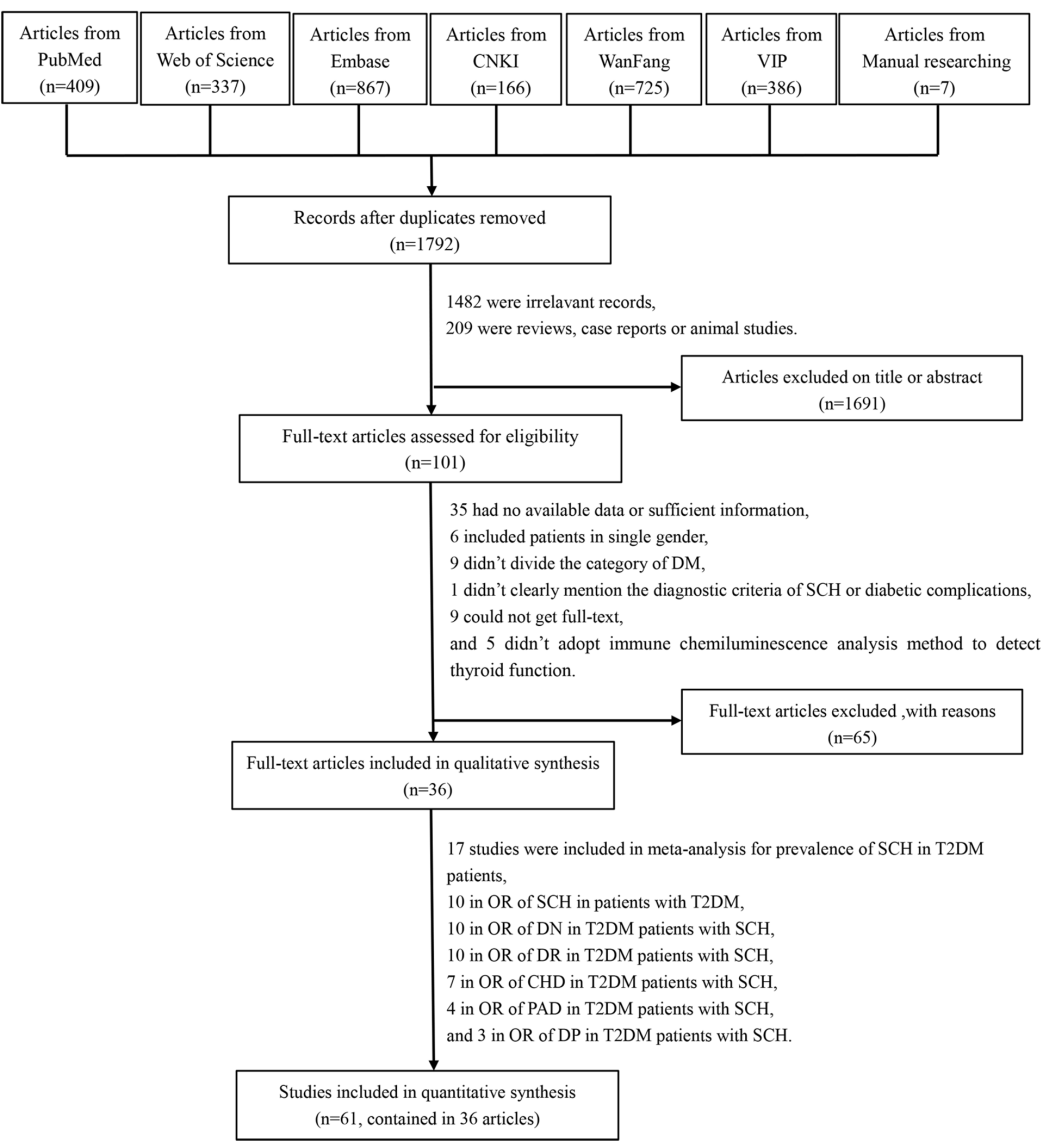
Flow diagram of literature search.

We identified 2897 citations in the primary search with 1792 records remained after duplicates were removed. Title and abstract screening removed 1691 studies, and 101 full articles were reviewed for potential relevance. These articles were screened based on the inclusion and exclusion criteria for the study. Finally, 36 articles containing 61 independent studies met our eligibility criteria. In our study, most of the included articles contributed to at least one meta-analysis. Of the 36 articles included, 17 studies assessed the prevalence of SCH in T2DM patients of different sexes [26–42], and 10 studies compared the prevalence of SCH in T2DM patients with non-diabetic patients [28, 31–34, 42–46]. Another 34 studies computed the risk of suffering from diabetic complications for T2DM individuals with SCH, 10 studies for diabetic nephropathy (DN)[41, 47–55], 10 studies for diabetic retinopathy (DR)[41, 47–51, 53–56], 7 studies for coronary heart disease (CHD)[48, 50, 55, 57–60], 4 studies for peripheral arterial disease (PAD)[48, 50, 55, 61], and 3 studies for diabetic peripheral neuropathy (DPN)[41, 50, 55].This detailed selection process is shown in Fig 1.

Study characteristics of the published studies that investigate the prevalence of SCH in T2DM patients are shown in Table 1. Features of researches comparing the possibility of SCH in T2DM subjects with non-T2DM are shown in Table 2. Next, information about the studies assessing the relationship between diabetic complications and SCH is shown in Table 3.

**Table 1:** Characteristics of the studies included in the meta-analysis for the summarized prevalence of SCH in T2DM patients.

First Author	Publication year	Area	Mean age^a^	Case(n)	Total(n)	Prevalence (%)	TSH	Research type	Score
Liu W [26]	2007	Beijing	59.3±14	127	1170	10.85	>4.0 mIU/L	CSS	5
Zhang NY [27]	2009	Jiangsu	60.4	50	416	12.02	>4.5 mIU/L	CSS	5
Ghazali SM [28]	2010	Nigeria	50.1±18.9	3	64	4.69	>4.31mIU/L	CC	4
Díez JJ [29]^b^	2011	Spain	F64.9±11, M62.2±11.2	34	318	10.69	>5.0 mIU/L	CSS	6
Deng X [30]	2011	Wujiaqu, Xinjiang	65	41	320	12.81	>4.5 mIU/L	CC	4
Zang SF [31]	2011	Hangzhou, Zhejiang	73.3±7.92	56	455	12.31	>4.94mIU/L	CC	3
Díez JJ [32]	2012	Spain	66.7±12	94	1112	8.45	>4.2 mIU/L	CC	6
Zhang MY [33]	2012	Nanchang, Jiangxi	56.3	15	120	12.5	>5.0 mIU/L	CC	3
Shi YS [34]	2012	Shenyang, Liaoning	55.5	40	413	9.69	>4.5 mIU/L	CSS	3
Wang Y [35]	2012	Xi’ an, Shanxi	60.4	36	208	17.31	>4.20mIU/L	CC	6
Liu XL [36]	2012	Xinyi, Guangdong	49.7±9.8	43	300	14.33	>5.0 mIU/L	CC	4
Sun LQ [37]	2013	Fuyang, Zhejiang	59.0 ± 7	23	140	16.43	>5.5 mIU/L	CC	5
Zhao R [38]	2013	Yinchuan, Ningxia	58±12	13	211	6.16	>4.20mIU/L	CSS	5
Xu XQ [39]	2013	Hefei, Anhui	46.35±11.93	22	164	13.41	>4.2 mIU/L	CSS	6
Xi WQ [40]	2014	Hohhot, Inner Mongolia	54.2±9.6	53	427	12.41	>4.2 mIU/L	CSS	5
Zhang DM [41]^b^	2014	Changsha, Hunan	C63,S60	244	1294	18.86	>5.6 mIU/L	CC	4
Zhu Y [42]	2014	Zhangjiagang, Jiangsu	61	25	246	10.16	>4.0 mIU/L	CC	4
**In total**				919	7378	10.2^c^			

a, Mean ages were expressed in mean ± SD or mean.

b, These original articles did not provide the mean age of the total population, so age data was extracted according to the age characteristics mentioned in the articles included.

c, adjusted pooled prevalence.

Abbreviation: F, female; M, male; C, subjects without SCH; S, subjects with SCH; TSH, thyroid stimulating hormone; CC, case control study; CSS, cross-sectional study.

**Table 2:** Characteristics of the studies included in the meta-analysis for comparing the possibility of SCH in T2DM subjects and non-T2DM.

First author	Publication year	Area	Mean age		T2DM (n)		Non-T2DM(n)		TSH	Research type	Score
			T2DM	Non-T2DM	Case	Total	Case	Total			
Gopinath B [43]	2008	Australia	68.8±7	67.6±7.6	4	113	17	950	>4.0 mIU/L	CC	5
Ghazali SM [28]	2010	Nigeria	50.1±18.9	53.8±24	3	64	0	36	>4.31mIU/L	CC	4
Zang SF [31]	2011	Hangzhou, Zhejiang	73.3±7.92	72.22±7.03	56	455	6	116	>4.94mIU/L	CC	3
Díez JJ [32]	2012	Spain	57.4 ± 16.1	66.7±12	94	1112	20	911	>5.0 mIU/l	CC	6
Zhang MY [33]	2012	Nanchang, Jiangxi	56.3	54.2	15	120	2	60	>4.2 mIU/L	CC	3
Zhang HY [44]	2012	Tianjin	57.7	58.9	26	180	5	160	>5.0 mIU/L	CC	3
Zhao FF [45]	2012	Qingdao, Shandong	60±12	60±12	250	2875	177	3612	>4.2 mIU/l	CC	3
Shi YS [34]	2012	Shenyang, Liaoning	55.5	54.3	41	413	4	120	>5.0 mIU/L	CSS	3
Liu C [46]	2014	Changsha, Hunan	60.4±10.5	61.5±9.3	25	190	9	180	>4.2 mIU/L	CC	3
Zhu Y [42]	2014	Zhangjiagang, Jiangsu	61	59.5	25	246	3	80	>5.6 mIU/L	CC	4
**In total** ^a^					539	5768	243	6225			

a, the adjusted pooled OR for SCH in T2DM was 1.93(95%CI: 1.66, 2.24).

**Table 3:** Characteristics of the studies included in the meta-analysis for assessing the relationship between diabetic complications and SCH. ^a^

First author	Publication year	Area	Mean age		SCH (n)		Euthyroid(n)		TSH	Research type	Score
			SCH	Euthyroid	case	total	case	total			
**Relationship between DN and SCH**											
Chen HS [47]	2007	Taipei	67.2±10.8	66.3±10.7	14	41	144	478	>4.0 mIU/L	CSS	10
Wang M [48]	2012	Hefei, Anhui	60.3±13.5	57.5±14.6	26	60	15	61	>4.78mIU/L	CC	5
Tang JD [49]	2012	Zhengzhou, Henan	64.6±10.2	58.4±9.8	106	164	537	972	>4.67mIU/L	CSS	5
Shen YJ [50]	2012	Wuhan, Hubei	62.2±10.6	59.8±9.8	93	148	138	300	>4.0 mIU/L	CC	4
Zhou JM [51]	2013	Yancheng, Jiangsu	59.8±12.3	58.6±13.2	31	48	104	227	>5.57mIU/L	CC	4
Furukawa S [52]	2014	Japan	63.7±11.1	61.1±11.3	6	36	23	379	>4.0 mIU/L	CSS	6
Lv B [53]	2014	Dalian, Liaoning	54.16±6.18	55.2±5.82	16	42	117	211	>5.0 mIU/L	CC	6
Lu L [54]	2014	Taiyuan, Shanxi	64±11	60±10	32	50	115	262	>4.5 mIU/L	CC	5
Zhang DM [41]	2014	Changsha, Hunan	63	60	150	238	454	952	>4.0 mIU/L	CC	4
Shao F [55]	2014	Changsha, Hunan	64±8.1	60.2±7.2	27	42	18	50	>4.0 mIU/L	CC	4
In total					501	869	1665	3892			
**Relationship between DR and SCH**											
Chen HS [47]	2007	Taipei	67.2±10.8	66.3±10.7	29	41	223	464	>4.0 mIU/L	CSS	10
Wang M [48]	2012	Hefei, Anhui	60.3±13.5	57.5±14.6	18	60	11	61	>4.78mIU/L	CC	5
Tang JD [49]	2012	Zhengzhou, Henan	64.6±10.2	58.4±9.8	101	164	509	972	>4.67mIU/L	CC	5
Shen YJ [50]	2012	Wuhan, Hubei	62.2±10.6	59.8±9.8	59	148	89	300	>4.0 mIU/L	CC	4
Zhou JM [51]	2013	Yancheng, Jiangsu	59.8±12.3	58.6±13.2	15	48	62	227	>5.57mIU/L	CC	4
Lv B [53]	2014	Dalian, Liaoning	54.16±6.18	55.2±5.82	16	42	85	211	>5.0 mIU/L	CC	6
Lu L [54]	2014	Taiyuan, Shanxi	64±11	60±10	15	50	73	262	>4.5 mIU/L	CC	5
Zhang DM [41]	2014	Changsha, Hunan	63	60	118	207	433	923	>4.0 mIU/L	CC	4
Chen Y [56]	2014	Wuwei, Gansu	74.56±3.11	75.74±4.21	1	33	21	267	>5.0 mIU/L	CC	6
Shao F [55]	2014	Changsha, Hunan	64±8.1	60.2±7.2	11	42	16	50	>4.0 mIU/L	CC	4
In Total					383	835	1522	3737			
**Relationship between CHD and SCH**											
Liu W [57]	2009	Beijing	58.3±14.2	61±13.7	41	127	45	200	>4.0 mIU/L	CC	6
Hong T [58]	2012	Wuhan, Hubei	63.2±10.6	59.5±9.8	14	53	35	286	>4.0 mIU/L	CC	6
Wang M [48]	2012	Hefei, Anhui	60.3±13.5	57.5±14.6	9	60	7	61	>4.78mIU/L	CC	5
Shen YJ [50]	2012	Wuhan, Hubei	62.2±10.6	59.8±9.8	32	148	103	300	>4.0 mIU/L	CC	4
Fu XL [59]	2013	Wuhan, Hubei	67.9±13.7	66.8±13.1	27	97	38	144	>4.94mIU/L	CC	5
Liu H [60]	2014	Zhuzhou, Hunan	61.7±6.8	59.9±5.2	22	52	53	276	>4.0 mIU/L	CC	3
Shao F [55]	2014	Changsha, Hunan	64±8.1	60.2±7.2	21	42	12	50	>4.0 mIU/L	CC	4
In Total					166	579	293	1317			
**Relationship between PAD and SCH**											
Wang M [48]	2012	Hefei, Anhui	60.3±13.5	57.5±14.6	7	60	8	61	>4.78mLU/L	CC	5
Shen YJ [50]	2012	Wuhan, Hubei	62.2±10.6	59.8±9.8	75	148	114	300	>4.0 mIU/L	CC	4
Duan Q [61]	2014	Beijing	58.67±10.7	54.67±8.93	21	62	8	78	>4.85mIU/L	CC	3
Shao F [55]	2014	Changsha, Hunan	64±8.1	60.2±7.2	24	42	20	50	>4.0 mIU/L	CC	4
In Total					127	312	150	489			
**Relationship between DPN and SCH**											
Shen YJ [50]	2012	Wuhan, Hubei	62.2±10.6	59.8±9.8	50	148	91	300	>4.0 mIU/L	CC	4
Zhang DM [41]	2014	Changsha, Hunan	63	60	147	230	389	940	>4.0 mIU/L	CC	4
Shao F [55]	2014	Changsha, Hunan	64±8.1	60.2±7.2	21	42	15	50	>4.0 mIU/L	CC	4
In Total					218	420	495	1290			

a: Diagnostic criteria for diabetic complications were provided in S1 Table.

All articles included have been assessed for quality control. For case-control studies, most presented moderate quality after evaluation with a NOS scale. For cross-sectional studies included, based on the recommended by AHRQ, the answers were “yes” for the majority of evaluation criteria. We did not rule out any article included in our meta-analysis according to their quality.

### Prevalence of SCH in T2DM patients

**Fig 2:**
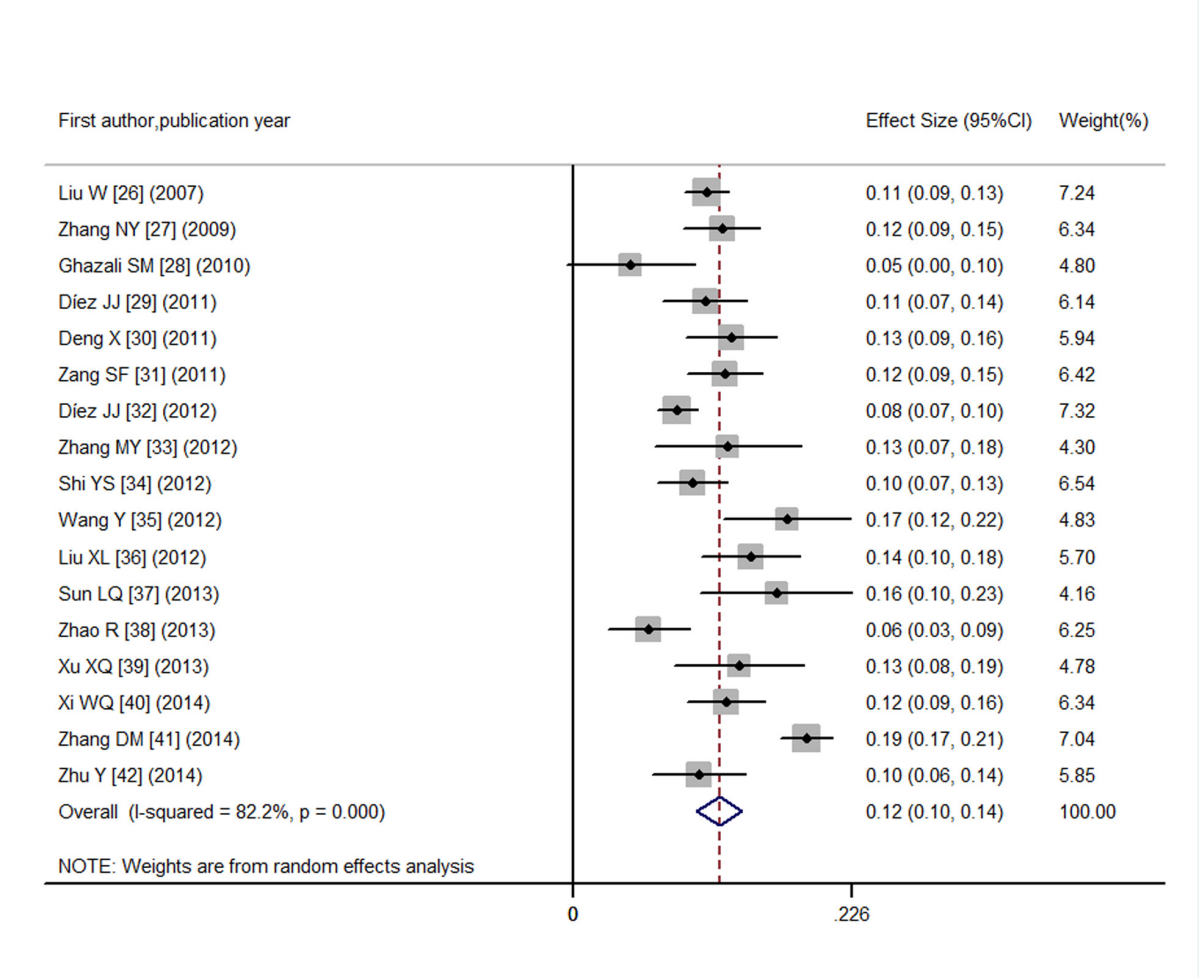
Prevalence of SCH in T2DM.

**Fig 3:**
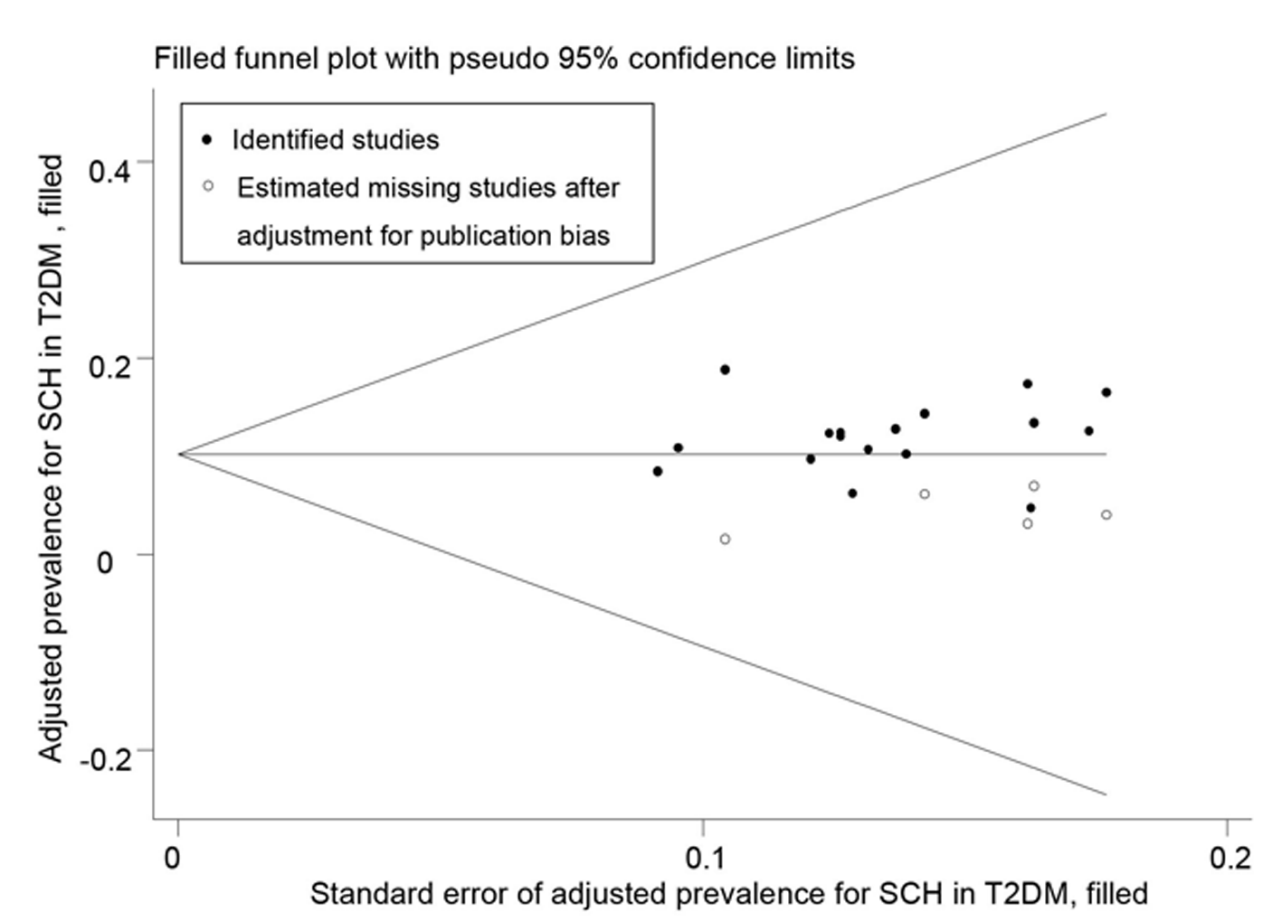
Trim and fill analysis for prevalence of SCH in T2DM.

This analysis contained data from 17 individual studies which included 919 cases and 7,378 subjects. Prevalence rates of SCH in T2DM patients ranged from 4.69% to 18.86% in the 17 included studies. There was significant heterogeneity among the chosen studies (I^2^ = 82.2%; *P* < 0.001), and the pooled prevalence of SCH in T2DM patients meta-analyzed using a random-effects model was 12% (95% CI: 10%, 14%) (Fig 2). However, publication bias was detected by visual inspection of funnel plot (S1 Fig) and the Egger’s test (*P* = 0.08). So trim and fill method was used to correct the result (Fig 3) and five potential missing data were replaced. The pooled prevalence resulted to be 10.2% (95%CI: 4.7%, 15.7%) after correction. As it is known that gender and age may influence the observed prevalence of SCH in non-diabetic population[9]. We subsequently conducted subgroup analyses to compute the prevalence rates according to gender, age, and location, trying to explain the heterogeneity. As shown in Table 4, we concluded that female T2DM participants had more SCH than male T2DM participants, and elderly T2DM patients (≥60 years old) were more frequently suffering from SCH. Also, we noticed that there were more SCH in Chinese T2DM, especially in Central China (prevalence of 18.9%), than in other countries that were included in our meta-analysis. Therefore, we found that the location might explain some of the heterogeneity.

**Table 4:** Prevalence of Subclinical hypothyroidism in patients with type 2 diabetes by different stratification factors.

Subgroups	Prevalence (95% CI)	No of study	Heterogeneity		Case/Total
			I^2^%	*P* value	
**Sex**					919/7378
**Female**	14.9 (12.4–17.5)	17	79.4	<0.001	593/3892
**Male**	8.8 (7.1–10.5)	17	63.9	<0.001	326/3486
**Mean Age**					919/7378
**<60 years old**	10.9 (8.8–12.9)	9	64.2	0.004	339/3009
**≥60 years old**	12.7 (9.7–15.8)	8	89.0	<0.001	580/4369
**Location**			-	-	919/7378
**Europe**	9.1 (7.1–11.0)	2	26.2	0.244	128/1430
**Africa**	4.7 (0.5–9.9)	1	-	-	3/64
**China**	12.6 (10.7–14.6)	14	79.5	<0.001	788/5884
East China	12.2 (10.8–13.6)	8	21.5	0.259	354/2919
West China	9.4 (2.9–15.9)	2	85.9	0.008	54/531
North China	11.0 (8.3–13.6)	2	37.3	0.207	93/840
South China	14.3 (10.4–18.3)	1	-	-	43/300
Central China	18.9 (16.7–21.0)	1	-	-	244/1294

**Fig 4:**
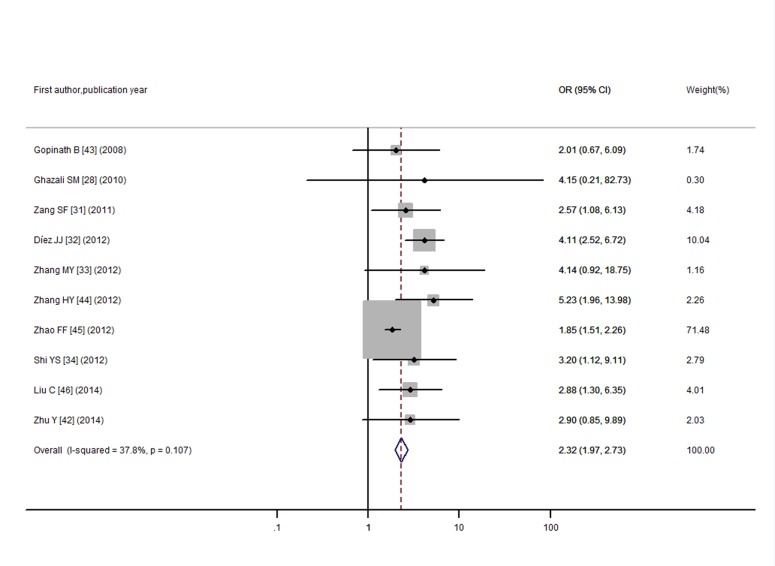
Odds ratio for SCH in T2DM.

**Fig 5:**
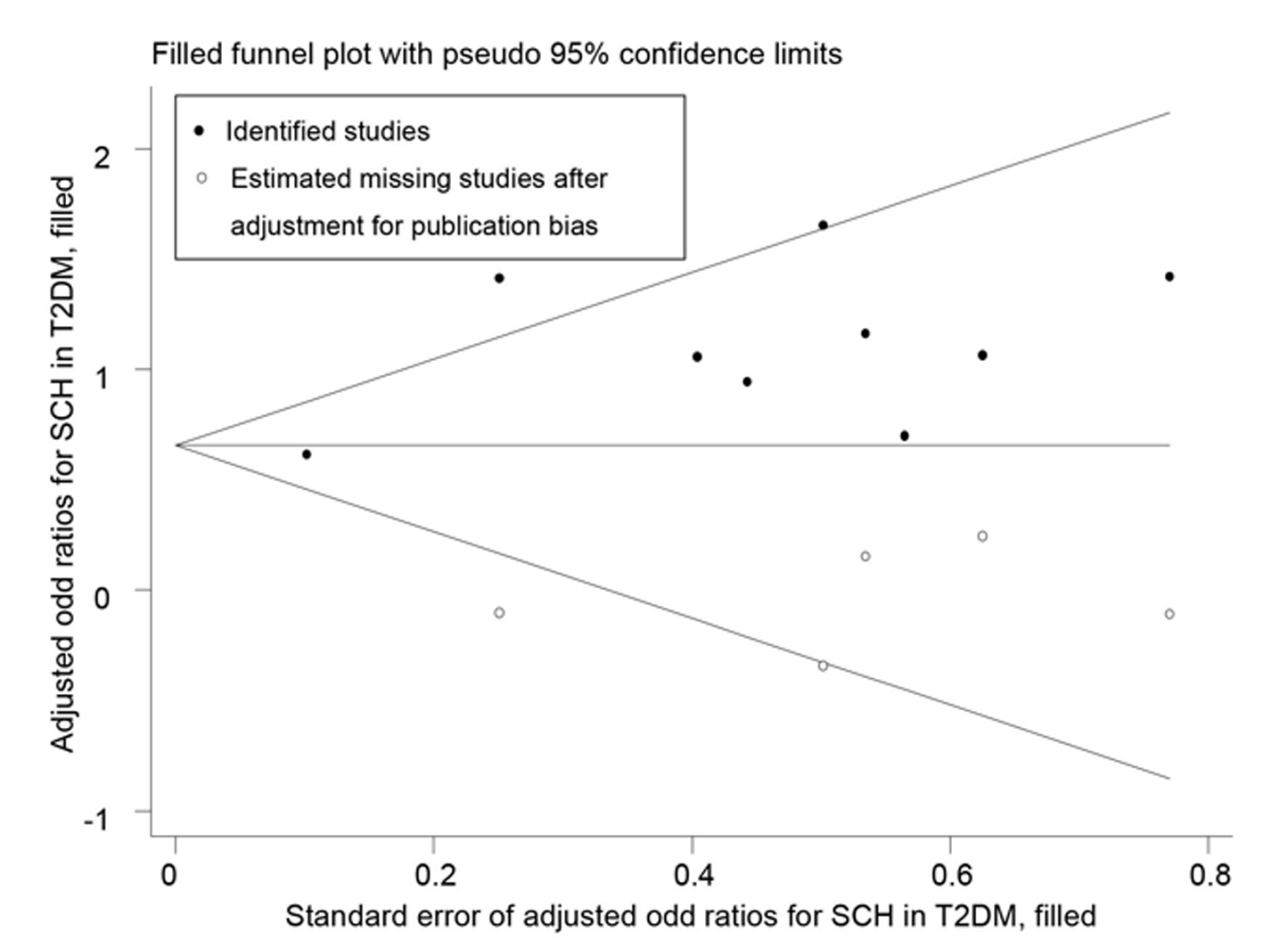
Trim and fill analysis for odds ratio of SCH in T2DM.

Furthermore, to find whether SCH was more prevalent in T2DM population when compared with non-diabetic subjects, we conducted a meta-analysis involving 5,768 individuals with T2DM and 6,225 individuals who were non-diabetics. There was mild heterogeneity among the 10 studies (I^2^ = 37.8%, *P* = 0.11).The OR of the meta-analysis for SCH events comparing T2DM individuals with non-diabetic individuals was 2.32 (95% CI: 1.97, 2.73) using a fixed-effects model (Fig 4). However, publication bias was detected by visual inspection of funnel plot (S2 Fig) and the Egger’s test (P = 0.04). Trim and fill method was applied to correct the result (Fig 5). Five potentially missing studies were replaced and the adjusted OR was 1.93 (95%CI: 1.66, 2.24).

### Prevalence of complications in T2DM patients with SCH

#### Diabetic microangiopathy

**Fig 6:**
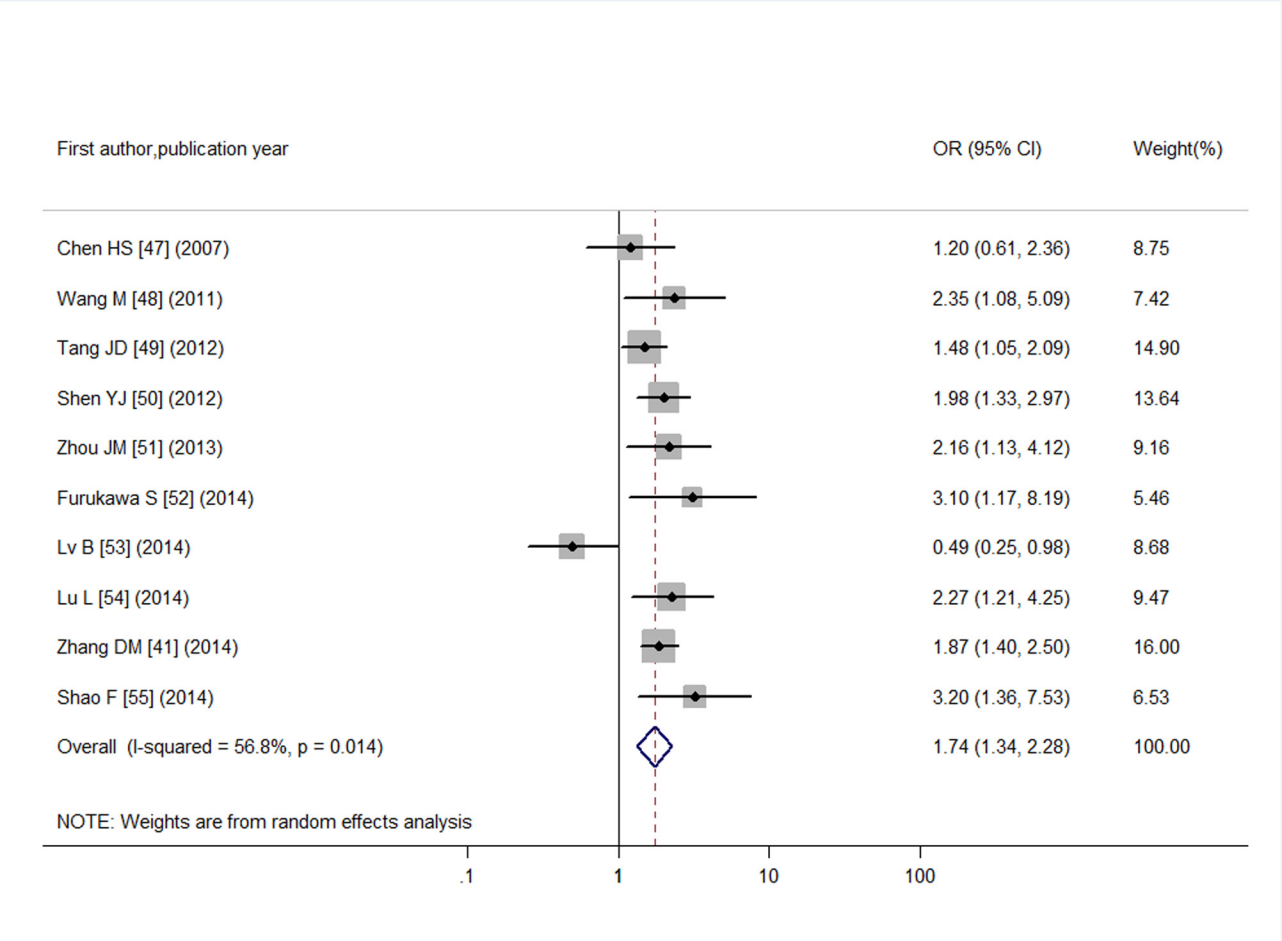
Odds ratio for DN in T2DM with SCH.

For DN, we enrolled in 869 T2DM individuals with SCH and 3,892 euthyroid T2DM individuals. Calculation of I^2^ index revealed evidence of moderate heterogeneity in the meta-analysis (I^2^ = 56.8%, *P* = 0.01). The pooled OR was 1.74(95% CI: 1.34, 2.28) using a random-effects model as shown in Fig 6. In the 10 articles enrolled in meta-analysis of association between SCH and DN, quantitative assessment using funnel plot (as shown in S3 Fig) and Egger’s test confirmed no statistically significant presence of publication bias (*P* = 0.84).

**Fig 7:**
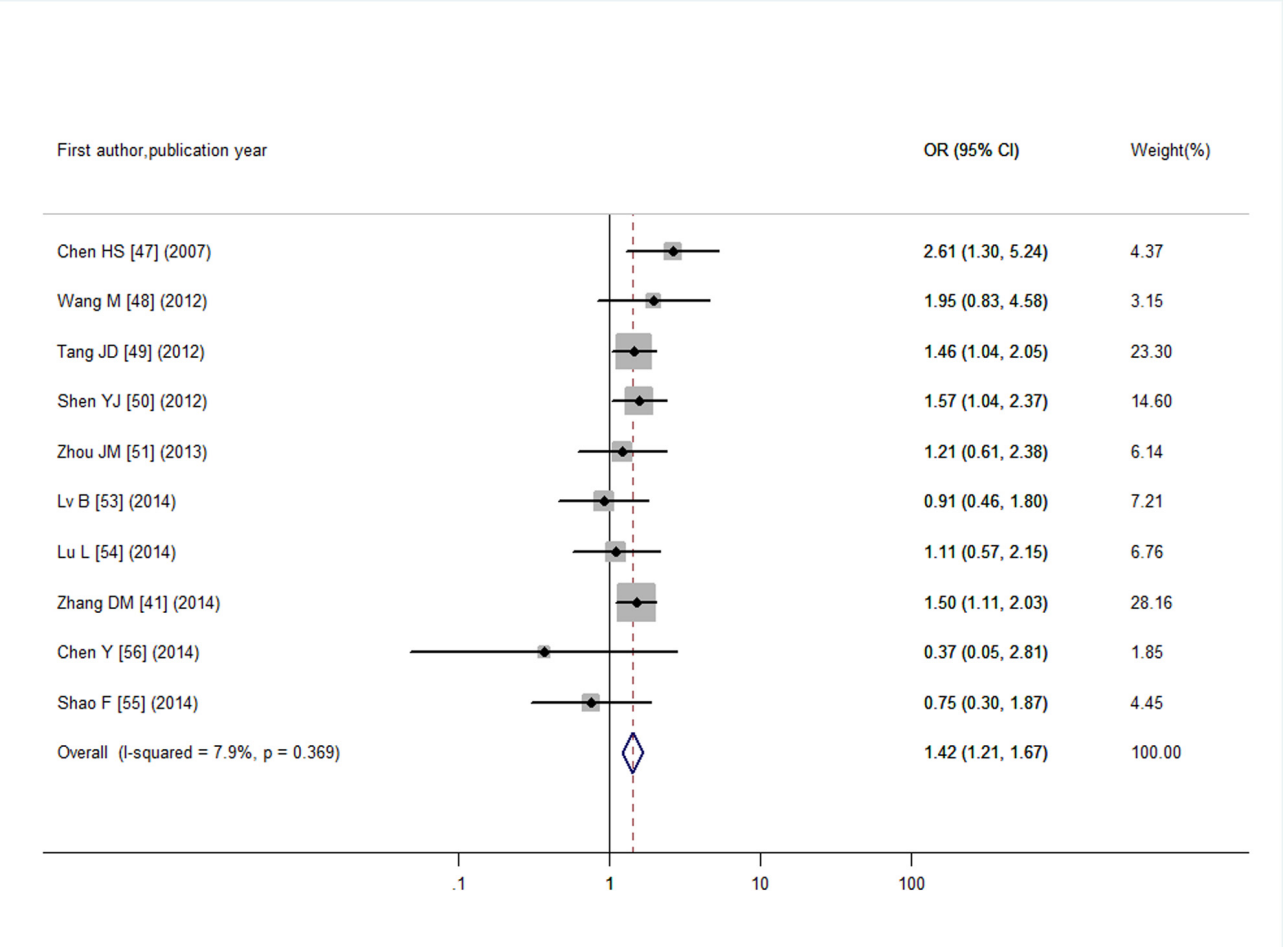
Odds ratio for DR in T2DM with SCH.

Meanwhile, in another 10 articles for DR, we enrolled in 835 T2DM individuals with SCH and 3,737 euthyroid T2DM individuals. I^2^ test indicated no obvious heterogeneity (I^2^ = 7.9%, *P* = 0.37) and the pooled OR was 1.42 (95% CI: 1.21, 1.67) calculated by a fixed-effects model (Fig 7). Funnel plot and Egger’s test suggested no considerable publication bias (as shown in S4 Fig and *P* = 0.21).

#### Diabetic macroangiopathy

**Fig 8:**
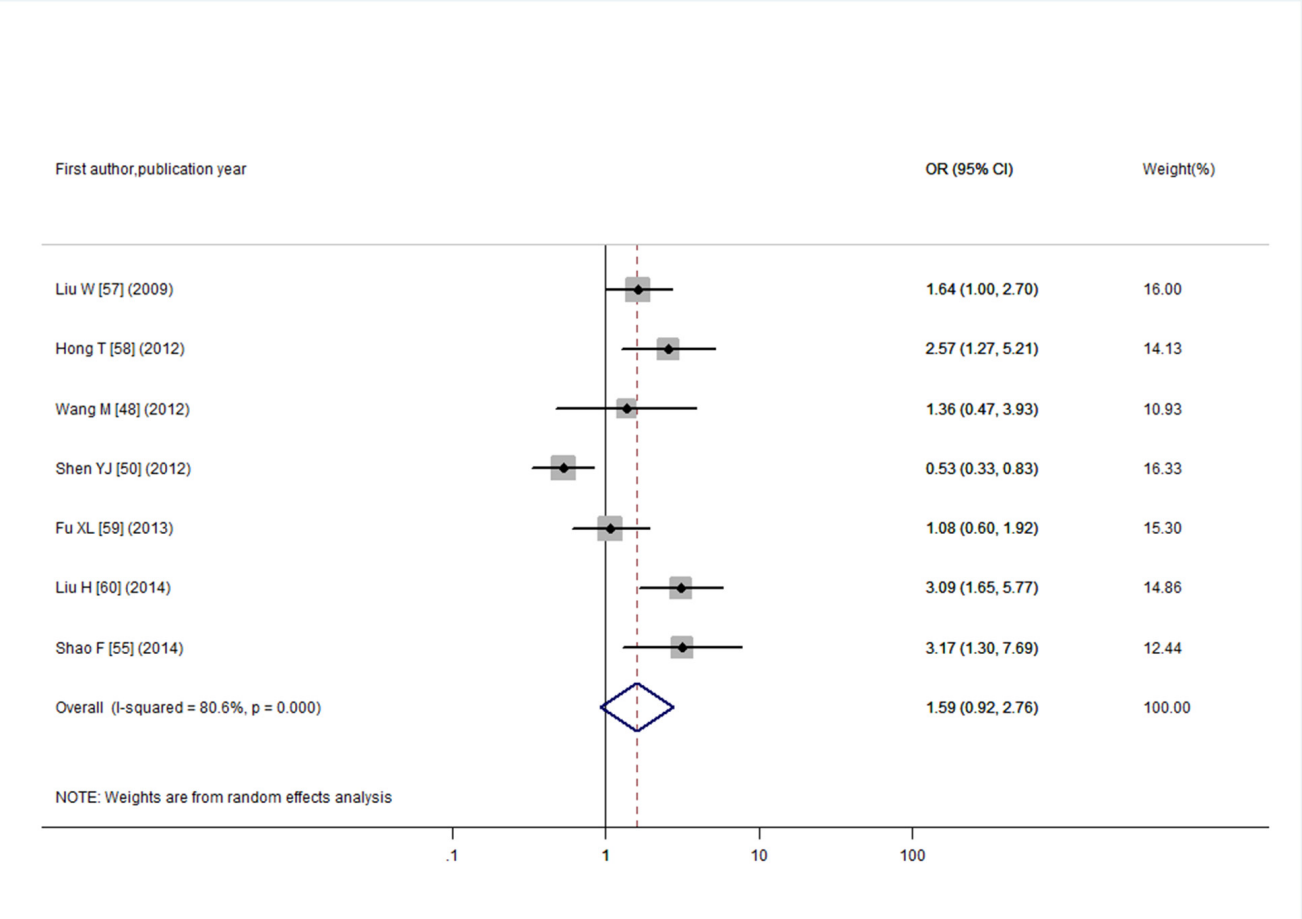
Odds ratio for CHD in T2DM with SCH.

To evaluate the association between CHD and SCH, we enrolled in 579 T2DM individuals with SCH and 1,317 euthyroid T2DM individuals in the meta-analysis. The included studies were significantly heterogeneous (I^2^ = 80.6%; *P* <0.001) as shown in Fig 8. The pooled OR was 1.59 (95% CI: 0.92, 2.76) by a random-effects model, which did not reach statistical significance. We found no publication bias according to funnel plot and Egger’s test (S5 Fig and *P* = 0.19).

**Fig 9:**
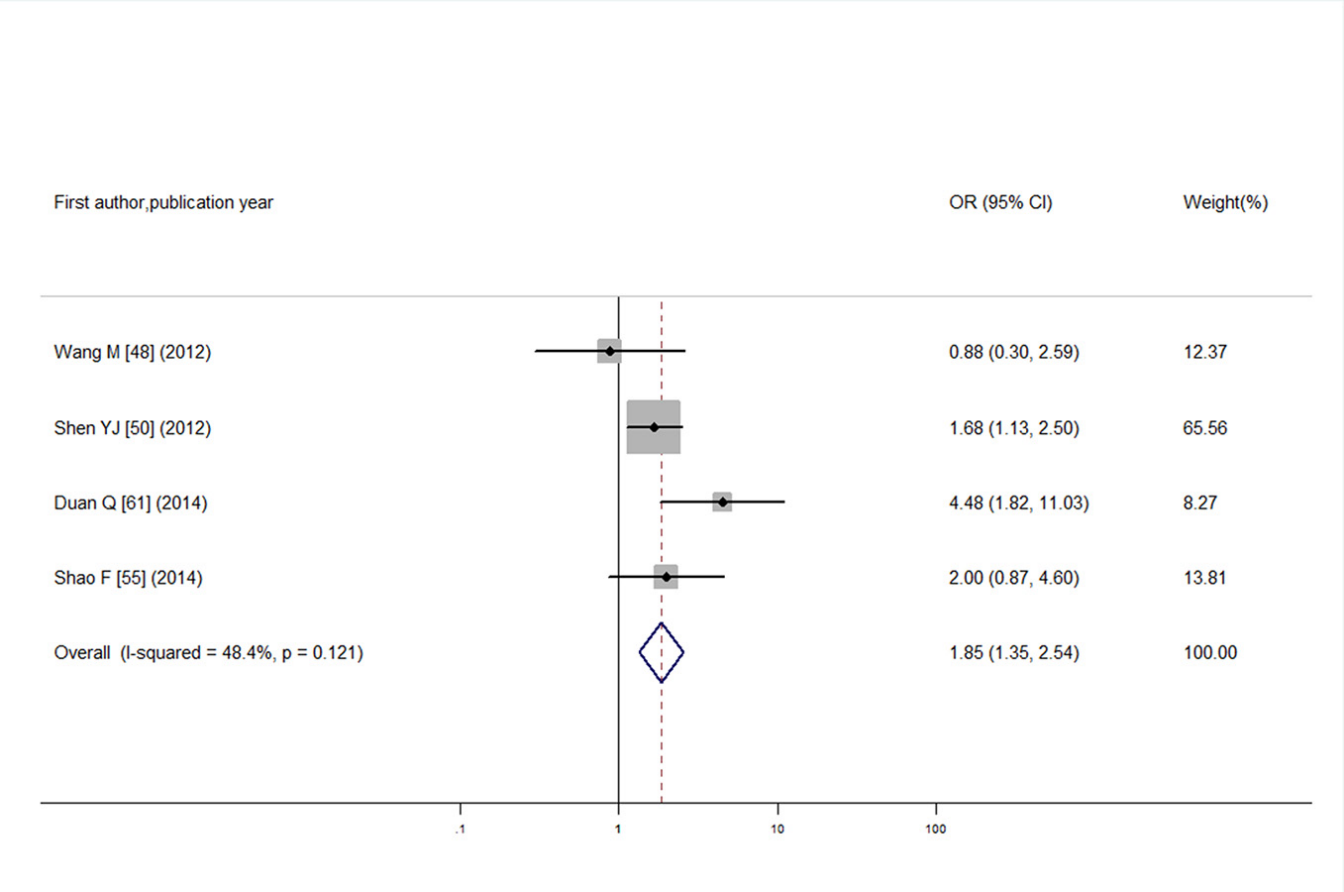
Odds ratio for PAD in T2DM with SCH.

For the association between PAD and SCH, we pooled data from 4 individual studies that included 312 cases and 489 controls. I^2^ test showed low heterogeneity (I^2^ = 48.4%; *P* = 0.12) and the pooled OR was 1.85 (95% CI: 1.35, 2.54) calculated by a fixed-effects model (Fig 9). No publication bias was observed according to funnel plot and Egger’s test (see S6 Fig and *P* = 0.83).

#### Diabetic peripheral neuropathy

**Fig 10:**
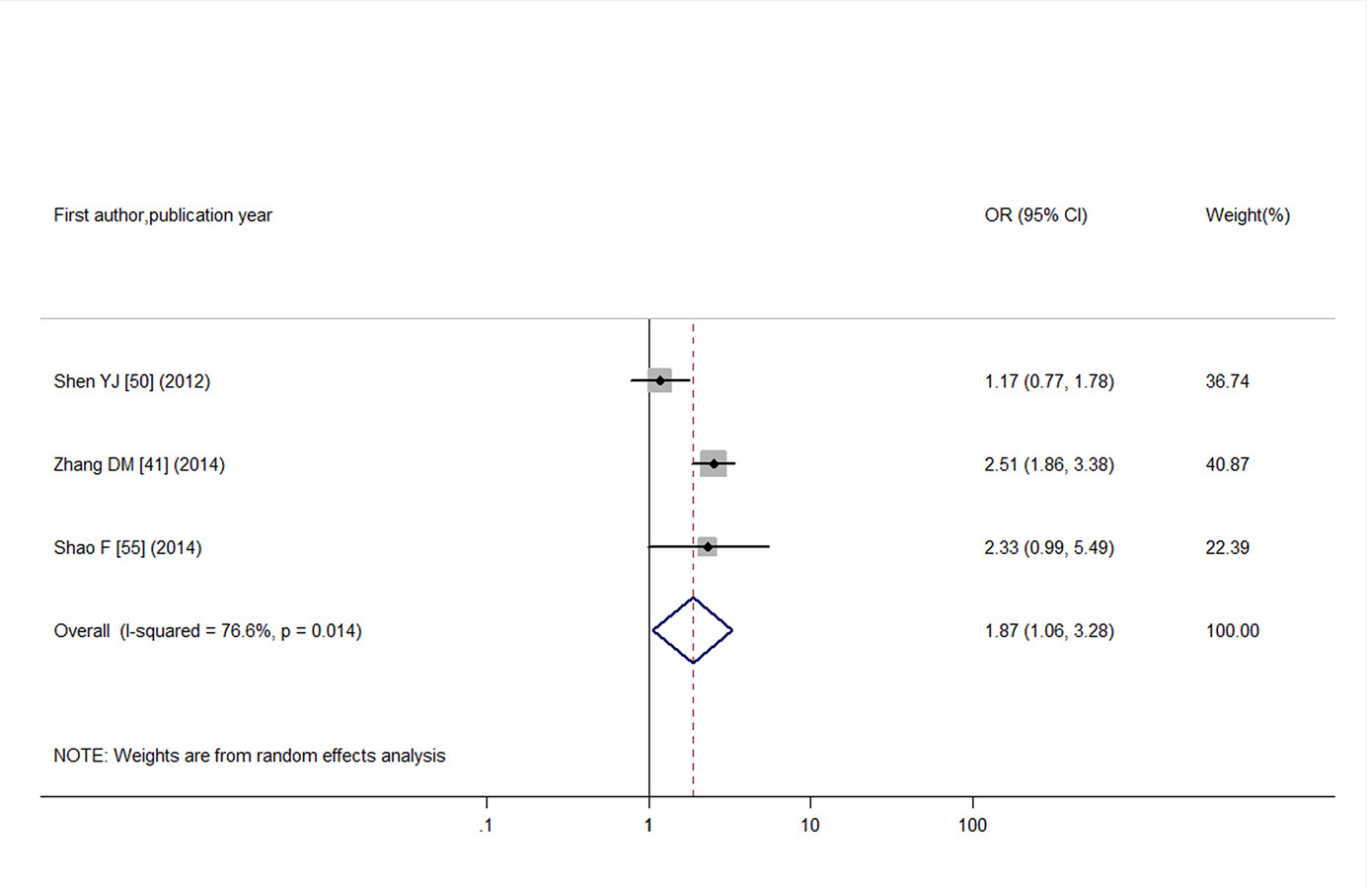
Odds ratio for DPN in T2DM with SCH.

For DPN, we conducted a meta-analysis consists of 420 T2DM individuals with SCH and 1,290 T2DM individuals who were euthyroid. I^2^ test showed significant heterogeneity among studies (I^2^ = 76.6%; *P* = 0.01). Analysis conducted with a random-effects model produced an estimated OR of 1.87 (95% CI: 1.06, 3.28), as shown in Fig 10. Funnel plot (as shown in S7 Fig) and Egger’s test (*P* = 0.84) did not confirm significant publication bias with a quantitative assessment.

## Discussion

We report that T2DM patients are more likely to have SCH when compared with general population (prevalence of 10.2%). Furthermore, when comparing patients with diabetic complications, we find that T2DM patients with SCH are more likely to have complications (such as DN, DR, PAD and DPN but not CHD). To our knowledge, this is the first meta-analysis to examine the relationship between SCH and T2DM and the association between diabetic complications and SCH.

The prevalence of SCH is 4–9% in the general population and our data suggests the same prevalence to be about 10.2% in T2DM population [9, 62–65]. As reported previously, factors such as age and sex may confound results of these meta-analyses [9]. Hence, subgroup analyses were performed. Gender difference, old age, and an apparent geographic disparity were observed to be related with the prevalence of SCH in T2DM. Specifically, there were 1.7 times more female SCH individuals than male in T2DM population, and T2DM individuals over 60 years of age were also more likely to experience SCH associated risks. These data agree with the NHANES Ⅲ study in non-diabetic population [9]. The prevalence also differed geographically. Diabetics with SCH were more common in mainland China than in Europe and Africa, with the highest prevalence occurred in Central China. However, the reason for this geographical difference remains unclear. Some researchers deem that genetic factors and environmental factors may lead to this phenomenon [22, 66], and further studies on this matter may be needed for confirmation.

The pooled prevalence of SCH was higher in patients with T2DM when compared with healthy controls (OR = 1.93) and the data was consistent with studies focused on overt hypothyroidism, which was more prevalent in diabetics than it was in the general population (OR = 3.45) [67]. The reason for having elevated TSH among diabetics is uncertain, for it may be due to a complex interdependent interaction. Previous studies suggested that leptin was greater in many diabetics [68, 69], which might stimulate synthesis of TSH by affecting the hypothalamic-pituitary-thyroid(HPT) axis via Janus activating kinase (JAK)-2/signal transduction and activation of transcription (STAT) 3 factor *in vitro* and *in vivo* [70]. Hyperinsulinemia was also prevalent in T2DM population and insulin might influence thyrotropin releasing hormone (TRH) and TSH when modulating glycemic status [71], thus, diabetics might have higher TSH.

We also concluded that SCH might aggravate diabetic microvascular complications such as DN and DR. Currently, patients with abnormal thyroid function may have decreased cardiac output [72], renal flow, glomerular filtration [73], and an increased peripheral vascular resistance [74, 75], and all of which can contribute to renal dysfunction. This may explain the higher prevalence of DN in diabetics with SCH. The mechanism behind the association between DR and SCH may be the decrease in circulating IGF-1, which is necessary for a normal retinal vasculature, and the decrease of IGF-1 may be mediated by thyroid dysfunction[76]. Further studies indicated that hypothyroid rats had significantly smaller and thinner retinas, with fewer dividing progenitor cells [77, 78]. In terms of macrovascular complications of diabetes, our data showed that the prevalence of PAD was higher in T2DM with SCH than in euthryoid T2DM. Hypothyroidism might increase the incidence of vascular disease in non-diabetic people, moreover, Monzani’s and Nagasaki’s groups reported an increased intima-media thickness (IMT) in patients with SCH [79, 80]. Kim and colleagues reported differences in total cholesterol, LDL-C, and mean-IMT between SCH and euthyroid participates [81]. Also, studies suggested that SCH might lead to vascular dysfunction with increased vascular stiffness and endothelial dysfunction which might contribute to PAD [82]. Hypercoagulable state and hemodynamic abnormality due to SCH might promote atherosclerotic progression which could drive forward PAD as well [83]. In non-diabetic population, CHD was associated with SCH [84], but our work did not confirm a similar association in T2DM population. Our present study observed that T2DM with SCH were more likely to have DPN and this was consistent with previous studies in non-diabetic models that confirmed an incipient axonal alteration presented in hypothyroidism, which might improve after hormone therapy [85]. Shirabe’s group suggested that changes in hypothyroid peripheral neuropathy correlated with segmental demyelination resulted from a basal metabolism disorder of Schwann cells [86]. Although numerous investigations have attempted to explain the mechanism of action between diabetic complications and SCH, more effort is required.

The rising prevalence of diabetes coupled with the increasing availability of sensitive thyroid function assays will lead more and more diabetics to be diagnosed with SCH[87]. Up to now, in terms of the screening policy, there is little consensus on thyroid disease screening strategies in routine T2DM care in the guideline of ATA and ETA [16, 17]. Our work may further provide additional evidence for formulating appropriate local public health policies and criteria. Also, metformin, a safe and effective biguanide oral hypoglycemic agent has been shown to reduce TSH in overt hypothyroidism and SCH [88]. Therefore, metformin may be suitable for T2DM population with SCH, as well as the early use of levothyroxine to prevent later complications.

Our study has several limitations. Firstly, the present meta-analysis was mainly based on cross-sectional and case-control studies, and these did not confirm causal relationship between SCH and T2DM or correlation between diabetic complications and SCH. Secondly, slight publication bias was observed in the meta-analyses of the pooled prevalence of SCH in T2DM and OR for SCH in T2DM, which might due to the limited numbers and the small sample sizes of publications recruited, plus some unpublished studies that were inevitably missed. What’s more, as much attention paid by Chinese academics to this topic, a large number of related researches emerge. At the same time, given the limitation of the researchers’ language ability, more Chinese articles were included, which could lead to a certain selection bias. In the subgroup analyses for the prevalence of SCH in T2DM, sample sizes of non-Asian population were relatively small, with no statistical power to explore the real associations, and the result of this should be explained carefully. Additionally, a part of published articles enrolled in our study were with poor quality. Lastly, in terms of ORs for diabetic complications such as CHD, PAD and DPN, articles enrolled were inadequate and the results might be coincidental. Based on the limitations above, more large-scale population-based prospective studies are needed.

In conclusion, our meta-analyses confirm that SCH is more prevalent in T2DM patients and that SCH may confer greater risk of diabetic complications. Thus, periodic thyroid function testing is necessary as a part of diabetic care. Finally, whether metformin for T2DM may ameliorate SCH in T2DM patients should be studied and whether individual levothyroxine intervention for SCH patients with T2DM may reduce or delay diabetic complications are of interest and worth studying.

## Supporting Information

S1 Checklist PRISMA 2009 Checklist. (DOCX)

S1 Fig Funnel plot of SCH prevalence in T2DM for included studies. (TIF)

S2 Fig Funnel plot of SCH for included studies. (TIF)

S3 Fig Funnel plot of DN for included studies. (TIF)

S4 Fig Funnel plot of DR for included studies. (TIF)

S5 Fig Funnel plot of CHD for included studies. (TIF)

S6 Fig Funnel plot of PAD for included studies. (TIF)

S7 Fig Funnel plot of DPN for included studies. (TIF)

S1 Table Raw data. (XLSX)
