# Supplementary figures and images for: Subclinical Hypothyroidism and Type 2 Diabetes: A Systematic Review and Meta-Analysis
Source: PLoS One. 2015 Aug 13;10(8):e0135233. doi: 10.1371/journal.pone.0135233 (PMC4535849; doi:10.1371/journal.pone.0135233)

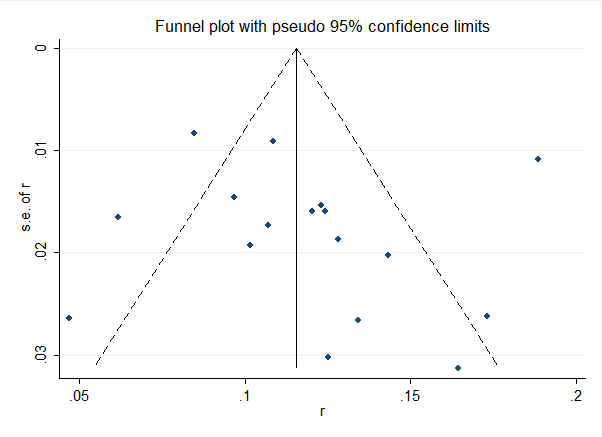

Supplement: S1 Fig — (TIF) [file pone.0135233.s002.tif]

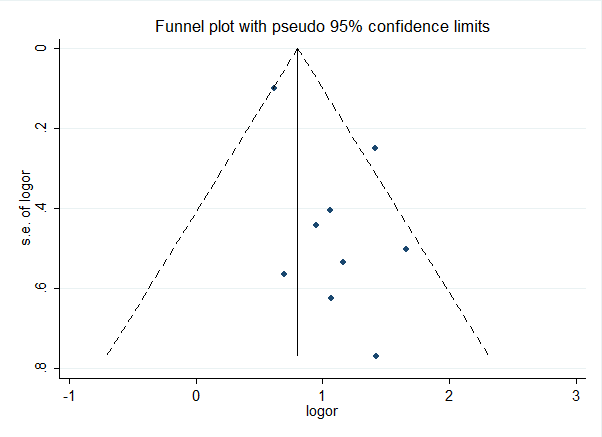

Supplement: S2 Fig — (TIF) [file pone.0135233.s003.tif]

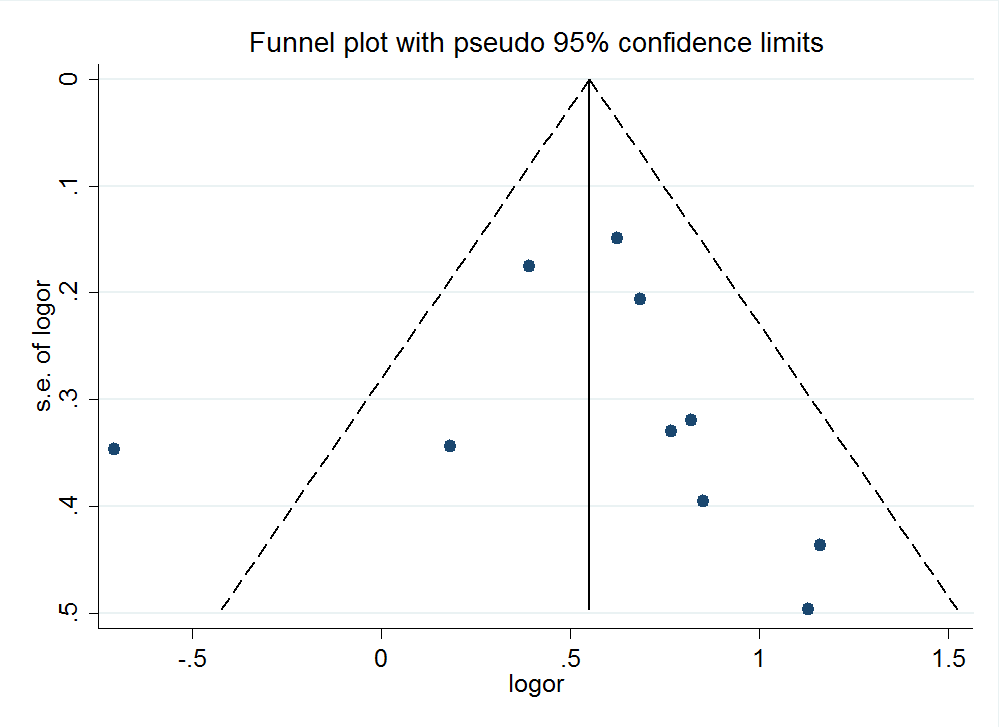

Supplement: S3 Fig — (TIF) [file pone.0135233.s004.tif]

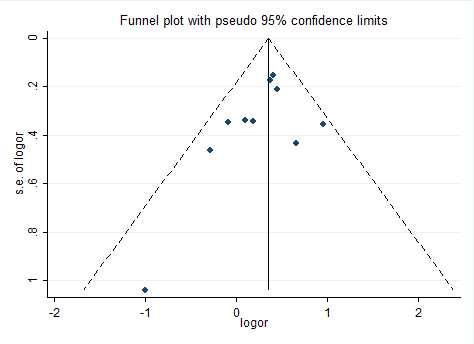

Supplement: S4 Fig — (TIF) [file pone.0135233.s005.tif]

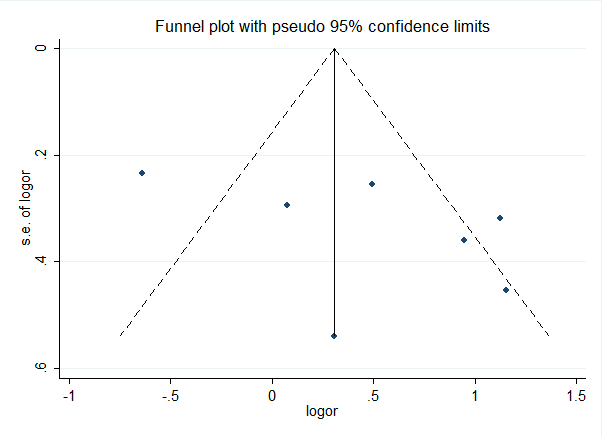

Supplement: S5 Fig — (TIF) [file pone.0135233.s006.tif]

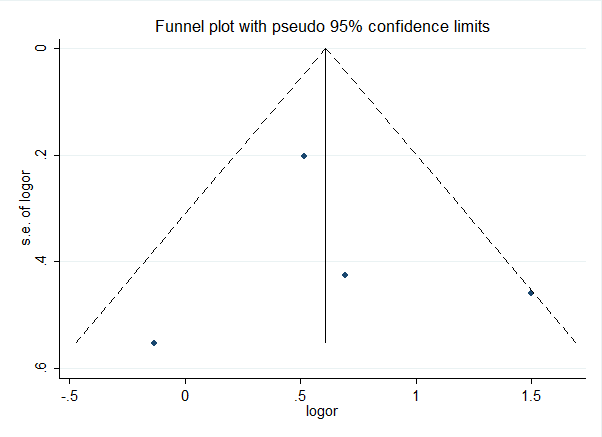

Supplement: S6 Fig — (TIF) [file pone.0135233.s007.tif]

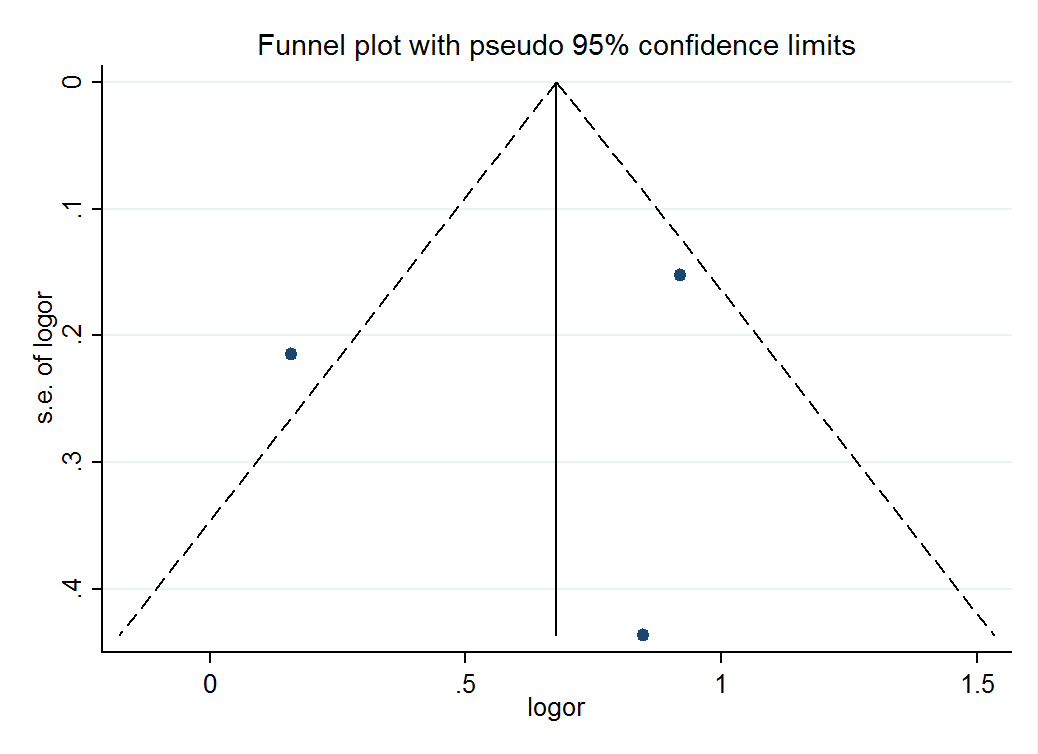

Supplement: S7 Fig — (TIF) [file pone.0135233.s008.tif]
